# Letter from Prof. J. P. White

**Published:** 1851-08

**Authors:** James P. White

**Affiliations:** London


### ART. III. — *Letter from Prof. J. P. White.*

London, *June*, 1851.

My Dear Doctor,—You will perceive that I am now in the great English metropolis, where I am passing a few weeks in visiting the hospitals and medical schools, as well as the crystal palace and other objects of interest which are here to be found on every side. Amid the occupations of sight-seeing and professional observation, I have hastily noted a few things which may prove of sufficient importance to warrant insertion in your valuable Journal. You will, I trust, excuse the want of arrangement which will be found, as I have attempted simply to write down a few facts in the same irregular manner as they fell under my own notice. In the first place, then, I would remark that through the politeness of Dr. Oldham, I have just been shown through the wards, lecture rooms, and museum, of Guy's Hospital. This is one of the largest and most venerable institutions of this great metropolis, and its reports have long been familiar to every medical reader. Its museum is among the richest in the world, and in it I was delighted to perceive the statues of many of those who have rendered themselves illustrious in medicine. Conspicuous among these stand that of Hunter, to whom every department of medical science is greatly indebted, and Jenner, who has placed under a heavy debt of gratitude every individual member of the human family who has already been, or shall be, for centuries to come, saved from that most loathsome of all diseases, small pox, by his humane substitute, vaccination.

In relation to the museum at Guy's, Bartholomew's, Middlesex, and University Hospitals, as well as in the great Hunterian museum, it is worthy of remark in passing, that great attention is paid to the preservation of *wet* morbid specimens. This is, perhaps, attributable as much to the want of artists in England who can copy in wax or otherwise, pathological appearances, as to the fact that these preparations of nature are supposed to be less likely to give erroneous impressions than the various productions of art. This example is worthy of imitation, as, by so doing, every practitioner, if he will make autopsic examinations, may aid in establishing a museum in his own vicinity, which in a short time would be ample for illustrating disease, and greatly contribute to the improvement of all who should have access to it. In Paris, where daily opportunities are afforded for examining recent specimens of disease, it is not so important that a collection of wet preparations should be made, and hence, perhaps, the reason why you there find little attention paid to this subject. In the colleges, cities, and even in the smaller villages in America, much might be done to advance the study of pathology, by concert of action among the various practitioners, in preserving and sending to one museum, open to all under proper regulations, every morbid product which comes within his own observation.—The following is the manner in which they are ordinarily put up, and which is, as you will perceive, simple and easily executed. Take a glass jar or vessel of convenient size and shape to accommodate the expanded specimen to be preserved, and fill with alcohol, diluted with one-third or one-half water, to such height as shall completely cover the object when arranged. In order to blanch the part to be preserved, and remove all the animal fluids, which, unless removed, will render the liquid opaque, it should be kept in water several days, changing the water frequently, and resorting to such gentle washing each time as is consistent with the nature and firmness of the tissues. Thus prepared, arrange the object in the vessel so that it can be distinctly seen without being removed or taken out. It may be suspended by a cord attached to a small stick nicely fitted to the inside of the top of the vessel, or the string may be carried through the first layer of bladder and then secured, or the cord suspending may be attached to another running horizontally over the top of the vessel, and fastened about its neck before it is sealed, or it may, in short, be suspended in any way which will not interfere with the perfect closure of the orifice of the vessel. A layer of moistened bladder is next carefully spread over this orifice, brought down upon the neck of the jar and fastened by a small cord carried firmly around it several times. Over this is next applied a layer of sheet lead or tin foil, and where great care is desirable, a plate or layer of each is preferable. It is well known that alcohol, especially after it has stood for a considerable time, contains an acid, hence if the lead be placed lowermost it would be acted upon, and the salt resulting from this combination will render the liquid turbid in addition to the destruction of the lead. Hence, the tin is placed next the first layer of bladder, or lowermost, to protect the thicker layer of lead, and between the two metallic layers a covering of solution of gum arabic, which, when dried, unites them, forming one plate of considerable firmness. Over this metallic surface is placed another coat of bladder, all being secured in the same manner as the first. The upper surface is now carefully painted and varnished, and the jar being then placed in an apartment where the variations of temperature are not great, will not ordinarily require farther attention for many years. I have been shown specimens at the museum of the Royal College of Surgeons, now in a state of perfect preservation, which are said to have been unopened for half a century. The constant attention which all wet preparations require when imperfectly put up, and the discouraging losses so often encountered, notwithstanding all our vigilance, has induced me to give you the detailed account of the method here generally pursued, and with entire satisfaction. In order to render the preparation generally and permanently useful, a history of the case should be carefully written out at the time, and the jar so labeled as to be able to refer to this description without difficulty.

But to return, without further digression, to Guy's Hospital. In accompanying Dr. Oldham, the distinguished teacher of obstetrics in that hospital, through the female wards under his supervision, many cases of great interest are pointed out. Without undertaking a detailed description of any, a brief reference to some of them may not be without interest. The first case claiming attention was one of ovarian dropsy. The patient was thirty-five years old, and the tumor, which now largely distended the abdominal parietus, had been first perceived three years ago, upon the left side. All therapeutic means having failed to excite the absorption of the effused fluid, and the abdominal distension having become so great as seriously to interfere with respiration, and impair the general health of the patient, tapping was deemed necessary. Accordingly the surgeon was called in, the abdomen again carefully examined by all present, and all the indications found present which ordinarily attend great abdominal distension with fluid. A large trochar was then passed in the usual manner in the lower part of the lined alba deep into the abdominal cavity. There now escaped two or three pints of hick ropy fluid, and notwithstanding all efforts to promote the farther discharge, the flow entirely ceased. The quantity removed, was not sufficient sensibly to diminish the abdominal distension, but it was found impossible to promote the farther flow from this orifice, and it was accordingly closed. This was doubtless a case of cystic dropsy, which is by no means infrequent, and which it is difficult, if not impossible, to diagnose before tapping.

Without a knowledge of the existence of such cases, the young practitioner, under similar circumstances, would be very likely to be considered either as having made a false diagnosis, or as having so unskilfully performed the operation for its relief as to have failed of obtaining the benefit which the patient ought to expect from it. The only course to be pursued when the fluid is ascertained, by puncture, to be confined in separate cysts, is either to close up the orifice already made, and then, or at some subsequent date, make a new opening in some part remote from the present, which may drain off the fluid, or pass some instrument through the orifice already made, in the hope of breaking down the partitions, thus permitting the escape of the fluid through the existing opening. The former course was in this instance most prudently adopted. The wound was closed by adhesive straps, a cordial administered, and the patient laid quietly in bed.

The next patient claiming attention, had fungous disease of the neck of the uterus, of a malignant character. The female was but about thirty years old, had had two natural labors, and could assign no cause which could satisfactorily account for the present disease. The general health was pretty good, and the appetite and strength unimpaired. The lower or posterior lip had suffered much more than the anterior, and presented one of the most perfect specimens of this form of malignant disease with which I have ever met. It bled freely whenever touched, and it was apparent, unless some measures were taken to arrest the progress of the disease, the life of the patient would soon be endangered. In view of all these circumstances, Dr. O. concluded to remove the diseased part with *potassa cum calce,* instead of resorting to the knife, which would most likely have been attended with frightful hemorrhage, or to the *fer rouge,* the measure so much in vogue with French surgeons for similar purposes, but which would alarm English or American women. The diseased parts were completely removed by means of this caustic without pain to the patient, or injury to the surrounding surfaces. In relation to the application of the heated iron above referred to, it may be added that frightful *as* its application certainly appears, I am satisfied that it is not attended with much pain when brought in contact with the mouth and neck of the uterus. I have been repeatedly assured by the patients themselves, after it has been freely applied by Cullerier and Jobert, that they suffered very little, or not at all, from its use. Believing that diseased action may however be modified or corrected by *potassa cum calce* — the acid nitrate of mercury, or the vienna paste, where the milder caustics are not sufficiently energetic, with nearly or quite the same certainty as with the hot iron, it will seldom, I apprehend, be found necessary to run counter to the prejudices of our countrywomen, by insisting upon its being substituted. It would at the same time be unfair not to admit that I have frequently witnessed the good effects resulting from the application of this seemingly terrific remedy. The cases most favorable for its application, are those in which the fibre of the uterus is relaxed, with a hemorrhagic tendency, and in which the milder caustics have been resorted to without modifying the local disease. Whether the same end could have been attained by a judicious application of the caustic used by Dr. O. on this occasion, and which removed the diseased parts with nearly the same precision as could have been done with the scalpel, is a question which can only be satisfactorily answered by reliable statistics carefully analyzed. For the present, my own limited observation impels me to the conclusion that the course pursued by the English practitioners will fulfil all the indications equally well, and that it is the practice which will be generally adopted in America.

Dr. Oldham, in the course of the same visit, introduced a patient with occlusion of the vaginal canal. The history of the case did not satisfactorily account for the firm adhesion here found between the opposing vaginal surfaces, and the complete obliteration of this canal. The woman had given birth to five children, the youngest being less than one year old, and the last labor, so far as the patient was aware, unattended by any circumstances which could have produced sloughing. A few months after, she began to have all the indications of menstruation, periodically returning with pain, but not attended with discharge. The severity of the pain increased with each return of these symptoms, and was accompanied with hypograstric enlargement and tenderness. She has severe neuralgic pains returning periodically during this menstrual effort as in dysmenorrhoea. Upon making an examination of the parts the vagina is found firmly closed an inch, or thereabouts, within the vulva, and the finger cannot break down these cicatrices by any force which it is prudent to make. The upper extremity of this canal was then easily brought into view and carefully examined, when it was found impossible to detect the smallest opening in this new partition across the vagina. The finger being carried into the rectum, was easily carried up to the uterus, which seemed enlarged as much as at the third or fourth month of pregnancy. The mouth of this organ could be detected occupying its natural position, and the neck undeveloped. It was ascertained, however, that the vagina above the cicatrix, was not distended; hence considerable doubt arose relative to the cause of the uterine development. If simply distended with retained menstrual fluid, why did it not descend into and fill the vaginal canal between the mouth of the uterus and the bridge or floor now closing this canal. It very rarely occurs that the neck of the uterus is obliterated, and although this organ presented all the indications of being filled with menstrual fluid, it was very certain none had escaped into the vagina. Under the circumstances it was deemed prudent to make a small opening through the rectum. This was easily effected by means of a narrow bistoury wound with lint, except at the point, and carried upon the finger up to the uterine globe, and then thrust through the walls of the uterus, when a free discharge of dark thick grumous blood left no doubt as to the cause of the distension.

The operation occasioned very little pain, temporary relief was obtained, the diagnosis established, and an opportunity afforded for undertaking some measure for permanent cure, with full knowledge of the difficulties to be encountered.

I might here mention a case of a similar character which was shown me a few days since, by the affable and erudite teacher of obstetrics in St. Bartholomews. In this instance inflammation and sloughing followed severe and protracted labor. The vesico-vaginal partition was destroyed to a very great extent, the bladder prolapsed into the vagina, and the urine dribbled away constantly, keeping the parts excoriated. The vaginal canal was closed above the fistula preventing the discharge of the menstrual fluid, if secreted, and what was still more unusual, the *urethra* had also been completely closed. By the most careful efforts it was found impossible, without force, to pass a small probe through this canal into the bladder. Before any operation could with propriety be undertaken for the purpose of closing the orifice in the vesico-vaginal partition, it is obvious that, as a preliminary step, it would become necessary to reopen this duct. The parts were at this time irritable and sensitive, the gentlest examination gave great pain, and all operative procedure was therefore postponed, and sedative and astringent applications to the parts directed.

Dr. West also showed me a case of ovarian dropsy, which, in consultation with Dr. Stanly, it was determined to resort to an operation (to me novel) for the permanent obliteration of the sac. The proposed operation was a modification of a suggestion recently made by a practitioner of Prague. They do not proceed upon the dangerous plan practiced with varying success upon the continent, of exciting inflammation in the walls of the sac, but undertake, by keeping it permanently evacuated, to produce contraction of its parietes and consequent obliteration of its cavity. To effect this end, it is proposed to tap the tumor at its most depending point, which can be easily reached through the vagina, and retain in the orifice a gum elastic catheter. A large curved trocher is to be first introduced into the tumor, which I could reach with difficulty with the finger in the vagina, the stilet then withdrawn, and after the contents of the sac shall have been discharged, pass up the flexible catheter through the canula. The catheter being much longer than the canula, the latter is easily withdrawn over the former, leaving it in the wound, where its external extremity should be secured by means of tapes to one of the thighs.

This operation will of course be applicable only to those cases where the general health is unimpaired, and where the disease has not made great progress. I shall watch with anxiety the result in the instance just referred to, and I cannot but indulge the hope that it will be found adequate to the cure of a proportion of those cases nearly all of which, notwithstanding the judicious use of therapeutic agents, sooner or later terminate fatally.

In connection with this subject, I will remark that the indefatigable Dr. Lee, in a pleasant interview which I had with him a few days since, showed me some statistical observations which he is preparing for publication, in relation to the operation for ovarian *tumor*, which I apprehend must lessen the frequency of its performance. He has collected one hundred and eight well authenticated cases of the operation in this country, and it may be added in most, if not in all of these, the operation was made by men of distinction. The results, as nearly as I can give them from memory, are as follows: in thirty-seven, or a little more than one-third of all the cases operated upon, the *diagnosis* proved incorrect, and after cutting down upon the tumor it was either found not to be *ovarian* or impossible to dissect it from its connections. Fourteen of these cases proved fatal. Of the remaining seventy-one in which the tumor was removed, twenty-four, or a little more than one-third, also died in consequence of the operation. Thus it will be perceived of the one hundred and eight operations, thirty-eight proved fatal› whilst forty-seven were benefited, and the remaining twenty-three were subjected to a painful and dangerous operation without any reasonable prospect of benefit. But I will not anticipate Dr. Lee's paper by enlarging upon this subject.

This able anatomist and obstetrician also presented me with a copy of a memoir on "the ganglia and nerves of the heart," which is to be accompanied with five engravings, all of which will be issued in a short time. Dr. Lee's memoirs on the ganglia and nerves of the uterus, published a few years since, together with the present discovery, are among the most important contributions to human anatomy, which have been made for many years. It is true that the correctness of these dissections and observations has been very bitterly contested, but the better opinion at this time is, that they are abundantly confirmed by other anatomists, and their existence is generally conceded.

In surgery I have seen more of Mr. Fergusson than any one else in London. His service is at King's College Hospital, where he lectures and where I have witnessed the performance of several operations, all of which were made with great coolness and dexterity. A few days since he cut for the stone in a very fat subject, and without the least apparent haste, completed the whole operation, extracting a stone of considerable size, in something less than two minutes. He made the lateral operation, using a knife or scalpel of his own invention, with which he divided the urethra and bladder, as well as the perineum. He succeeded in retaining the fluids in the bladder by plugging up, as it were, with the thumb of the left hand, the orifice made in the bladder, until the incision was finished, when being permitted to escape suddenly, it brought the stone directly into the upper orifice of this new outlet, and it was seized with extraordinary facility. This operation is now seldom made in the Parisian hospitals. By the various improvements made upon the original method of Civiale, both by himself, who continues to operate at Hospital Necker, and the younger surgeons, they succeed by persevering and repeated efforts in perforating and crushing, and thus in the end bringing away through the urethra the largest calculi. It often requires repeated efforts, bringing away but small portions each time, with such delay and treatment during the meantime as shall allay the vesical irritation, but by gentleness and patience the object may almost always be thus accomplished, and the stone discharged without resorting to the knife when it can be reached. In the instance alluded to it was deemed too large for crushing, and lithotomy judged most safe. Certain it is that the operation was admirably performed, and the patient, I may add, is convalescing, being the last time I saw him, nearly cured.

I have seen Mr. F. operate upon several cases of hare lip. In double hare lip, with the intermaxillary substance projecting as it sometimes does, he makes two operations, contenting himself in the first, with raising the integument and exciting this projecting ossific and cartilaginous substance, leaving the operation for approximation of the parts for another occasion. The hemorrhage from the bone thus divided, is sometimes troublesome, and was in this instance arrested by the application of the heated iron. He still uses long steel needles for bringing and retaining the parts in contact, clipping both ends of the pin after insertion, precisely as made in America many years since. Mr. Fergusson promotes the union of the parts brought in contact, and relieves the tension upon the needles by means of an apparatus invented by himself, with which he makes permanent pressure upon the cheeks. The instrument consists of a circular band of flat steel made like that used for making trusses, which is passed around the posterior part of the head with the anterior extremity nicely padded and applied to the cheeks, thus giving firm support to the soft parts, and preventing their retraction and straining upon the pins when the child cries. This band, which passes below the occipital projection, is retained in place by straps which, by the aid of buckles, may be made of any desired length, and which run over and anterior to the head. When there is great retraction of the parts, he applies this apparatus some days before he subjects the child to the operation, with a view of approximating the surfaces to be united. It certainly seems to answer the purpose for which it is designed much better than straps running anterior to and over the lip operated upon, and without pressing injuriously upon the parts held in contact by the stitches or pins. Its use may be continued for some time after the removal of the pins, to prevent the parts recently united from being torn asunder by any sudden effort of the child, made before the cicatrix has become sufficiently firm to be relied upon.

Mr. Fergusson introduced a case of prolapsus *ani*, of long standing, the bowel protruding to great extent, with every effort to defecate, and which had resisted all efforts for its permanent reduction. The patient could, by straining, bring down a large globular mass of thickened and engorged tissues. The sphincter muscle had been so much dilated by the long continued protrusion of this mass that it seemed to have lost its tone or contractile power, and when the tumor was reduced by carrying it above this muscle, it did not possess the power necessary to retain it there. Mr. Fergusson treated this case by cutting out large folds of the integument in several points around the margin of the anus. The excisions diverged from the line of junction between the mucous membrane and the external skin, being broadest at the point most distant from the centre.

This operation certainly seems well calculated, by the extensive cicatrization which would result, to contract the projecting mass, and Mr. F. assures me that, unlike what I should have expected, it never leaves a hardened eschar, or ring, about the anus, which would seriously interfere with the proper performance of the functions of the sphincter muscle. Mr. F. next made the operation for fistula in *ano*, reiterating the old, but judicious direction, that the internal orifice should be carefully sought and included in the operation, else it would be very likely to prove a source of irritation, and occasion the necessity of a second resort to the knife.

In amputations of the thigh, all the English surgeons, so far as I have seen, make the double flap operation, much as described and practiced by Mr. Liston; whilst Roux, and some of the other French surgeons, still practice the circular. After cutting off the thigh of a young musician, which was rendered useless by paralysis, a patient was introduced whose middle finger had suffered from whitlow, with the second phalanx now carious. The finger was removed by surgeon Patridge, at the metacarpal joint, although the last joint of the finger might have been preserved. Mr. P. remarked that the joint, if left, would be a perfectly useless stump, would interfere often with the use of the hand, and besides, would be a conspicuous deformity. Whereas, by amputating above, the hand contracted without the unsightly stump to indicate the loss, and the missing finger would scarcely be remarked.

All the operations of magnitude, and indeed where neither the danger to life, or the pain to which the patient would be subjected seemed very considerable, which came under my observation, were performed under the influence of anæsthetic agents, ordinarily chloroform alone being used. It is variously administered, upon the handkerchief simply, or with a great variety of apparatus, according to the taste or fancy of the operator. In London it is not only administered more frequently for the performance of operations than in Paris, but is given so as to bring the patient much more completely under its influence, and this impression is in the former also continued until the operation is completed. There seems, on the part of the surgeons here, to be entertaned no apprehension of immediate or ultimate injury from the full and continued use of anæsthesia; whilst the Parisian surgeons yet regard it with suspicion, and use it with much greater caution.

The older surgeons, Sir Benj. Brodie and Mr. Lawrence, are still actively engaged in professional pursuits. Sir Benj. declines operative surgery, but is daily consulted by a large number of patients attracted here from all parts of the globe by his wide-spread reputation. Mr. Lawrence, notwithstanding his ample fortune, and immense private business, continues his connection with Bartholomew's Hospital. He has one of the most beautiful places in England, *Eeling Park*, five miles from the centre of the most fashionable part of London. It contains sixty acres, and is laid out into parks and gardens, in the highest state of cultivation, and tastefully ornamented with arbors, walks, arches, fountains, and statuary. I am credibly informed that the annual cost of maintaining, in their present condition, the green-houses and grounds, alone exceeds three thousand pounds sterling. It should also be added of Mr. Lawrence, that he is the embodiment of English hospitality, and is especially attentive to members of the profession who may visit London. I have met at his table at the same time, representatives of the profession from France, Belgium, Hanover, and Prussia, which is but a fair illustration of his generous hospitality. Regretting that you will be compelled to read so long a letter without more to interest,

I remain, your friend,

JAMES P. WHITE.

